# Home working during the COVID-19 pandemic: The experience of drug and alcohol support workers

**DOI:** 10.1177/22799036251381226

**Published:** 2025-11-12

**Authors:** Nigel Lloyd, Wendy Wills, Imogen Freethy, Olujoke Fakoya, Charis Bontoft, Jaime Garcia-Iglesias, Suzanne Bartington, Gavin Breslin, Neil Howlett, Julia Jones, Katie Newby, Nigel Smeeton, Amander Wellings, David Wellsted, Katherine Brown

**Affiliations:** 1 School of Life and Medical Sciences, University of Hertfordshire, UK; 2 CRIPACC, School of Health & Social Work, University of Hertfordshire, UK; 3 Institute of Applied Health Research, University of Birmingham, UK; 4 Bamford Centre for Mental Health and Wellbeing, Ulster University, UK

**Keywords:** home working, work from home, COVID-19 pandemic, drug and alcohol support services, substance use, teleworking, public health, remote working

## Abstract

**Background:**

Drug and alcohol support workers play a vital role in addressing the growing burden of substance-related harm and mortality. The COVID-19 pandemic led to an abrupt and significant shift towards home working for many in this workforce. This study explores these workers’ home working experiences, addressing a research gap and providing valuable insights for staff, organisations delivering public health services, and service users.

**Design and methods:**

This qualitative study explores home working experiences of 30 drug and alcohol support workers in northern England during the COVID-19 pandemic. Data collection included innovative digital methods: (1) digital timelines (*n* = 16); (2) in-depth interviews (*n* = 17); (3) five focus groups (*n* = 12). Timeline text was treated as qualitative text data. Interviews and focus groups were recorded, transcribed, and coded. Data were subject to Framework Analysis.

**Results:**

Seven themes were identified: (1) Difficulty balancing and separating work and home life; (2) Importance of setup, infrastructure and conducive work environment; (3) The move to remote/home working – a process; (4) Convenience and efficiency benefits; (5) Loss of the social: – reductions in social connectedness and feelings of isolation; (6) The importance of the ‘office’ for connection, communication, socialising, and information sharing; (7) Managing remotely – the development and implementation of strategies and ways of coping.

**Conclusions:**

While home working offers some benefits for substance use support workers, providers, and service users, it also introduces significant challenges. Understanding these is critical for service optimisation. A hybrid (in-person/remote) deliver model, combining home and co-located, office-based working may be optimal.

## Significance for public health

The COVID-19 pandemic necessitated significant changes to the working practices of many public health practitioners, including the requirement for some to work from home. However, there is a lack of research into public health employees’ experiences of home working. This study provides a novel insight into the work from home experiences of those who provided drug and alcohol support during the COVID-19 pandemic and the challenges and benefits home working presented. It highlights the ways in which the experiences and needs of home working public health staff may differ from those working from employers’ premises, and the implications for effective service delivery and the future integration of homeworking into public health service delivery models. The study focuses on those delivering substance use services, but findings have broad relevance for commissioners and deliverers of public health services.

## Introduction

The COVID-19 pandemic and subsequent social distancing mandates and guidance resulted in an unprecedented increase in the numbers of employees working from home rather than from their employer’s premises. Between 2019 and 2020 in the European Union, for example, the number of employed people aged 20–64 years who usually worked from home more than doubled from 5.5% to 12.3%, with a further increase in 2021 to 13.5%.^1^ In the United Kingdom, the number of homeworkers more than doubled between late 2019 and early 2022 following the March 2020 request by the UK Government for people to work from home where possible.^2^

Although many who worked from home during the pandemic subsequently returned to their workplaces on a full-time basis, others did not, and increased home working has remained a part of working life for a significant proportion of UK workers.^3^ Data from Great Britain indicate that, as of Spring 2022, the majority of those who began home working because of the COVID-19 pandemic planned to continue with at least some home working in the future.^4^ Between September 2022 and March 2023, 44% of workers reported home or hybrid working,^5^ and recent data suggest that in 2025, the levels of home and hybrid working remain similar.^6^

Industrial sector and the type of duties required by an individual’s job are the factors most associated with working from home,^7^ with some sectors and roles lending themselves to home working more readily than others. In the UK, it is estimated that more than 80% of those in the IT, finance and insurance sectors worked from home in January 2021, compared with approximately 20% of those in industries such as the wholesale, retail, or food service industries, and 40% of those in the ‘human health and social work activities’ sector,^7^ which includes public health services.

Health and care services such as drug and alcohol support are among those less suited to home working due to the relational nature of this work,^8^ typified by a focus on collaborative work with service users, developing trust between service users and the organisations and staff supporting them, and supporting service user autonomy and choice.^8,9^ Thus, while for workers in some sectors, the increase in home working brought about by the COVID-19 pandemic reflected an extension or formalisation of the home working they already periodically engaged in Milasi et al.,^10^ for those in health and care roles, home working is likely to have been a completely new experience. Child and family support, primary healthcare, mental health services, and drug and alcohol support, were among the services that experienced reconfiguration, and a previously unprecedented move to home working during the pandemic.^11,12^

In England, local authorities are responsible for the delivery of key public health functions, including drug and alcohol treatment services. Prior to the COVID-19 pandemic, face-to-face service delivery and in-person interaction between staff and those in receipt of drug and alcohol services was a cornerstone of this provision, as was access to recovery services through locality-based health or community settings.^13^ The move to home working for those supporting service users’ recovery was therefore a significant change, the implications of which have yet to be fully understood. This study explores the ‘working from home’ experiences of drug and alcohol support workers required to do so because of the COVID-19 pandemic. It aims to offer insights into how home working impacted the lives and working practices of those delivering drug and alcohol services and highlights the implications for staff, organisations, service delivery, and those in receipt of services.

Since working from home is likely to continue to some degree for drug and alcohol support staff, albeit to a lesser extent than during the height of the pandemic, it is important that their home-working experiences and its implications are understood and inform policy and practice. Existing studies explore the work from home experiences of various practitioners involved in the delivery of public health services during the COVID-19 pandemic, including social workers,^14^ clinical psychologists,^15^ and mental health support workers.^16^ While there are broad similarities between drug and alcohol support and other health and care services – such as the relational nature of work and the shift towards remote modes of delivery during the pandemic – recent studies have highlighted the diversity and complexity of workers’ COVID-19 home working experiences.^17,18^ These studies underline how role-specific factors, established ways of working with service users, and contextual factors - such as sector, organisational culture, and service expectations – can shape the experience of working from home.

To meaningfully inform future drug and alcohol service delivery, it is crucial to develop sector-specific, occupational, and service-level insights into the home-working experiences of support staff. This study makes a significant contribution by focussing specifically on the unique and situated experiences of drug and alcohol support workers during the COVID-19 pandemic.

Moreover, among those studies that have examined the home-working experiences of health and care professionals, few have treated home working as their primary focus. Notable exceptions include Harry and Brady’s^19^ study of emergency ambulance staff and McChesney et al.’s^20^ study of workers in a cancer treatment centre. More commonly, home working analyses are part of broader investigations into the effectiveness of telehealth^21^ or form part of analyses of service user experiences of the wider impacts of the pandemic on services.^16^ In contrast, the current study places the home-working experiences of drug and alcohol support workers at its centre.

While the extant literature has much to contribute, the need for a specific focus on the experience of drug and alcohol workers is clear. To our knowledge, no studies have yet explored the home-working experiences of those delivering drug and alcohol support services during the COVID-19 pandemic – a gap that is particularly significant given the current service provision context. In England and Wales, for example, drug- and alcohol-related mortality rates are on an upward trajectory. The number of drug-related deaths registered in 2022 was the highest since records began,^5^ and there is evidence that treatment outcomes were affected during the pandemic, with an increase in deaths among individuals in drug and alcohol treatment (OHID, 2023b).^22^ Further, modelling of the long-term impacts of pandemic-related changes in alcohol consumption suggests serious future consequences. For instance, Angus et al. (2024)^23^ project substantial increases in alcohol-related hospital admissions and deaths over the next 20 years, with significant implications for health service capacity and resourcing. Together, these trends point to an urgent and growing need for effective, resilient, and well-supported drug and alcohol services, at a time when public sector budgets are under pressure. Understanding staff experiences of service delivery, which include their experiences of home working, is therefore critical to informing and optimising future service delivery.

We understand this study to be the first qualitative study that focuses specifically on the home working experiences of those charged with delivering drug and alcohol support services and it therefore makes a unique contribution to the substance use support and broader public health literature.

## Methods

The data presented here were collected as part of a larger mixed-method evaluation of remote delivery of drug and alcohol support services during the COVID-19 pandemic across a large metropolitan area of Northern England.^24^ This broader study aimed to understand the impact of service reconfiguration on services, staff, and service users, with the goal of informing the future optimisation of service design. We included specific questions on home working in the staff interviews and focus groups to contextualise participants’ circumstances, and the interviews reinforced that home working constituted a distinct and highly salient aspect of staff experience that warranted dedicated analysis and separate reporting.

A manuscript presenting qualitative findings from our wider study is currently under review.^25^ While that study provides an overview of pandemic-related service delivery changes, particularly the shift to remote provision and its impact on service users and quality of care, the present article focuses solely on staff experiences of home working – a topic not analysed or reported elsewhere as part of the wider study.

The current is article draws on findings from the analysis of qualitative data collected from staff delivering drug and alcohol support services. We utilised three data collection methods: interactive digital timelines, focus groups, and individual in-depth interviews. Participants were invited to take part in at least one, and up to all three methods. Data were collected between March and June 2021. All data were collected remotely, with participants taking part during their regular working hours, either from their workplace or while working from home.

The study took a broadly phenomenological approach^26^ to capture the common features of the phenomenon under study. Our qualitative analysis is underpinned by a broad constructivist/interpretivist orientation^27^ which acknowledges the role of individuals’ experiences and interpretations in framing their subjective, constructed realities. The consolidated criteria for reporting qualitative research (COREQ)^28^ have been used as a guideline for our reporting.

### Recruitment and sampling

In the research study locality, an organisation (henceforth referred to as the ‘lead organisation’) delivered treatment services on behalf of the local authority, in partnership with various public and third sector organisations. Services offered included information and advice; harm reduction; health screening; one-to-one intervention via a support worker; mutual aid sessions; opioid substitution treatment; psychosocial interventions; detoxification; and relapse prevention support. In March 2020, the lead organisation began reconfiguring support services in anticipation of pandemic-related social distancing requirements. In the period that followed, many aspects of service delivery moved from in-person to remote delivery and most staff were required to work predominantly from home.

For this study, existing staff members at the lead organisation and two partner organisations were recruited via internal email lists. These organisations employ approximately 170 staff, with the lead organisation accounting for about 150 of these. Approximately 3000 people are typically supported each year by these organisations. An email invitation, composed by the study team, with accompanying text from service managers, was circulated to staff. Staff volunteered to participate by providing basic details (e.g. work contact details and job role) and written informed consent to participate via Research Electronic Data Capture, REDCap.^29^

All those registered to participate at the start of timeline data collection (32 of the approximately 170 staff members) were invited to complete an interactive digital timeline,^30^ with 16 ultimately doing so. Similarly, all 47 participants who had registered by the time focus group recruitment began were invited to take part in a focus group. For interviews, maximum variation sampling^31^ was used to obtain a diverse sample, with the aim of exploring a range of experiences. Within sample size constraints, we aimed for variation in terms of job roles and specialisms, time in post, seniority, and type of substance use supported by the staff member.

### Participants

Thirty-six staff members participated in the wider study. All were aged 18 years or older and were staff members or volunteers working directly for, or in partnership with, the lead organisation or one of the service providers to deliver drug and/or alcohol treatment services. Of the initial 36 staff members, 30 indicated that they had worked from home at some point since the start of the COVID-19 pandemic. Experience of home working during the pandemic was an inclusion criterion, and therefore, only data from the 30 participants meeting this criterion were included in the analysis presented here. Of these, 15 had some management responsibility (sometimes in addition to front-line delivery duties), 14 had front-line service delivery but no management role, and one had an administrative-only role. Median time in post was 5 years and 3 months. Sixteen participants completed an individual digital timeline, 12 took part in a focus group, and 17 took part in an individual, in-depth, semi-structured interview.

### Procedure

#### Digital timelines

Timeline data collection took place during March 2021. Participants were emailed a link to an online whiteboard platform, Lucidspark,^32^ which presented a linear timeline with milestones from the start of ‘lockdown’ in March 2020 to the date of timeline completion. Such timelines have been shown to facilitate detailed participant reflections on past experiences.^30,33,34^ Participants were asked to complete their individual timeline by adding work-related and personal experiences of delivering drug and alcohol services during this period. They were given 10 days to complete the timeline during which they were able to revisit the timeline an unlimited number of times to make additions or amendments.

#### Focus groups and interviews

Between April and May 2021, five focus groups were conducted. Each involved between two and eight (Some focus groups included individuals who had not worked at home during the COVID-19 pandemic) participants, ran for between 60 and 100 min, and was facilitated by two researchers.

Seventeen individual, semi-structured, in-depth interviews were conducted between May and June 2021. Interviews, conducted by a single researcher, lasted between 40 and 90 min. Data collection focussed on the nature and effectiveness of service delivery reconfiguration, implementation of remote service delivery, participants’ experiences of changes (including home working), and learning for future service delivery.

Focus groups and interviews were conducted remotely via videoconferencing with participants’ permission to audio/video record.

### Data coding and analysis

The audio from videoconference recordings of interviews and focus groups were transcribed by a General Data Protection Regulation (GDPR) compliant transcription service to produce verbatim transcripts. Online timeline text was treated as qualitative text-based data and, as with the interview and focus group transcripts, was coded using NVivo 12 software (20).

Following transcription, data were analysed using Framework Analysis^35^ involving the following staged process: familiarisation with the data; coding; development of an analytical framework; applying the analytical framework, charting, and mapping and interpretation.^36–38^

Six members of the research team (NL, KB, OF, IF, JIG, CB) reviewed a subset of transcripts to inductively develop an initial list of codes. A series of meetings between these team members was then held to discuss codes and review further transcripts, with the aim of iteratively developing the codebook and analytical framework. Between these meetings further inductive and deductive coding was conducted. Deductive coding utilised codes derived from interview and focus group schedules and the research questions. NL and OF then developed and drafted an initial codebook and analytical framework which was refined and agreed by the remaining team members.

Seven members of the team’s Public Involvement in Research group (PIRg members are lay people who support the research process and offer expertise through lived experience of heath service use) received training in framework analysis and reviewed the codebook and an anonymised transcript to offer suggestions for improving its clarity and usability. PIRg suggestions resulted in the refinement of code names and descriptors.

Three research team members (NL, OF, IF) then continued with transcript review until consensus was reached regarding the final codebook. The same team members coded all transcripts, with further codebook refinement as necessary and any coding discrepancies agreed through consensus. NL, OF, and IF were then responsible for the charting, mapping and interpretation processes, led by NL. These involved creating a framework matrix spreadsheet for focus group and interview data, with a separate spreadsheet used to organise timeline data by framework topics. NL began the mapping and interpretation process by reviewing both spreadsheets to generate a preliminary list of potential themes. This list was then shared with IF and OF, who also systematically explored the matrix, identifying themes within and across framework categories. Through regular meetings and iterative discussion, the team reached consensus on a final list of themes, with ‘thematic saturation’^39^ reached prior to ceasing our analysis.

## Results

Seven themes were identified: (1) Difficulty balancing and separating work and home life; (2) Importance of setup, infrastructure and conducive work environment; (3) The move to remote/home working – a process; (4) Convenience and efficiency benefits; (5) Loss of the social: – reductions in social connectedness and feelings of isolation; (6) The importance of the ‘office’ for connection, communication, socialising, and information sharing; (7) Managing remotely – the development and implementation of strategies and ways of coping.

### Theme 1: Difficulty balancing and separating work and home life

The difficulty staff had in separating and balancing work and home life when working from home was commonly expressed. Participants described the need to balance the demands of work and home without the physical and psychological separation that leaving home to go to work had previously provided. This often meant that working life impinged practically and emotionally on personal lives. Leaving work behind at the end of the working day became more difficult.

If you’re in the office you’ve got your colleagues around to debrief with, and possibly as you’re walking away from the office you can leave all that behind. When you’re at home if you’ve absorbed that all day there isn’t actually a safe space to go, for you to get rid of your feelings, and how that’s left you feeling. (FG4) (FG denotes focus group; INT denotes interview (with number indicated))

When you’re working at a location, you’re getting in the car, you’re driving and you’re going there, you’re doing your work. You’re switching off on the drive home, and then you’re home and you’re sort of different to what you were when you were at work. (INT 11) (FG denotes focus group; INT denotes interview (with number indicated))

Participants described difficulties disengaging from working life when working in a home environment. They described feeling that home working led to working life intruding into what was previously a private space or working hours extending into what would previously have been personal time. Working for longer periods of time by starting earlier or working later was a commonly cited example.

The computer is still sat there, that even though I did do all those things that you’re supposed to do, like, pack it up and put it away, move everything back. Yeah, it just - I’d still be thinking work, I’d still be maybe opening up emails later on at night. It did - it did emotionally, make work stretch into longer periods of my life. (INT 4)

Longer days for home workers, starting earlier and finishing later. Increase of meetings due to virtual platforms. (Timeline entry referring to September 2020).

Staff commented that supporting service users in treatment for substance use can sometimes require them to engage in conversations that broached sensitive topics, were emotionally charged, or were upsetting or distressing. Some participants found it challenging to hold such conversations and interactions within their home environment. For them, these sensitive, work-related discussions were now being brought into their personal living space. They suggested that what had previously been a haven from work was now sometimes intruded into by upsetting or distressing interactions.

I recall having a conversation with someone and he just shouted at me on and on and on about something that we couldn’t get sorted for him and I just thought “wow, not in my kitchen mate!” [Laugher] So that was just, that was bizarre. (FG 5)

For some participants, the residue of those upsetting experiences appeared to remain in the personal spaces in which they had occurred.

This is a difficult job and you have some very quite difficult conversations. . .and you’re in your home having those conversations with people, and then you don’t leave that environment. So all that is still there, and you don’t have that separation, as such. (INT 8)

### Theme 2: Importance of setup, infrastructure, and conducive work environment

Some staff described the challenges to home working caused by a home environment or home-working setup that was ill-suited to home working. These included regular interruptions by others in the household; difficulty conducting personal or sensitive conversations with service users because of a lack of private space or the presence of other members of the household; inappropriate workstation setups leading to back pain; and a lack of the necessary IT infrastructure (e.g. adequate Wi-Fi).

So I did some comprehensive assessments with people over the phone, and even that I found difficult because you’ve got a young child at home that’s not going to allow you to sit through a comprehensive assessment with a client, without making a noise. . .. (INT 2)

I usually work on the dining room table, or on the sofa, which isn’t good for your back, so you end up with a bad back as well working from home. (INT 10)

Staff also described changes that they had made to their home environments over time to provide a setting in which they could work effectively. These included procuring new equipment or furniture, setting up dedicated work areas within the home, and adjusting when they conducted work tasks (e.g. phone calls and meetings) to avoid distractions or provide privacy.

I think over time it [home workstation setup] was starting to take a physical toll on my lower back, and what have you. So I’ve got a little table that I sit at here, where I’ve got myself a stand and I’ve got myself a big monitor. . .so that’s more comfy, and I’ve got a proper chair now. But, yeah, I think the working environment was the bit that was harder. (INT 1)

There were times that doing Zoom video calls and things like that were just not appropriate while I’m at home because my [detail removed] kid would probably come barging in at some point. . . I was quite strict about kind of not doing it if they [children] were at home. It just wasn’t appropriate. (FG5)

### Theme 3: The move to remote/home working – A process

A prominent theme in participants’ descriptions of working from home was that the shift to home working was experienced as a process of adaptation and change, particularly in relation to working practices and managing work and home life. The move from traditional, office-based working to home working had been an abrupt and unexpected one and staff described needing to adapt. It was initially a novel experience that they became used to, and typically more at ease with, over time.

It felt a little bit weird. Like ringing clients from home felt a bit strange, that you were speaking to somebody, and someone else is upstairs, or do you know? You’re in your own kitchen kind of thing, and it felt a bit. . . But I think we soon got used to it, (INT 18)

Participants described the newness of their home-working scenarios, and few had been well prepared for the experience. Staff talked about a process of reflection, learning, and adaptation that took place as they gained more experience of home working and of what did and did not work well for them. They described adapting both work and home life so that each did not overly impinge on the other.

We’ve had a reshuffle in the house, so my [child] has gone upstairs, my [other child] has gone into the other room, and I’ve turned this room, what was [his/hers], into an office. . .all this coming and going from people coming in and out the house, was distracting as well, when you’re trying to do Zoom meetings, and all that kind of stuff. So at least now I’m out the way. (INT 12)

There’s that temptation there to maybe log in to emails a little bit earlier in the morning, or a little bit after you finish just to catch up on things. Whereas in the office you wouldn’t do that as much, so I’ve had to be quite disciplined and not do that. . .I had to set firm boundaries for myself quite early on. (INT 9)

### Theme 4: Convenience and efficiency benefits

In describing the benefits of home working, participants commonly referred to the increased efficiency and convenience afforded by working from home. For example, they described the time saved by not needing to travel to and from their employer’s premises or service users’ homes.

And it’s a strange thing, because talking to a woman on the phone instead of going out to see her, saves you a lot of time. So rather than having to drive across [city removed] to see your client and have a chat with her and then drive back again, you’re calling her. (INT 5)

I’d say probably time was the main thing, like being able to just have more time that is my own, rather than travelling. (INT 3)

Participants also highlighted the benefits of home working for allowing them to attend meetings with colleagues more easily because the need to travel had been removed.

I know that you’re not in the room and you can’t see people, but I think being able to attend was so much better, because it’s easier just to. . .You’re not having to travel to sit in a room and have the same conversations. So I think that was much better. (INT 1)

Other participants stated that home working allowed them to work more efficiently as they could better focus on work tasks without the interruptions that might occur when working in an office with colleagues.

Working from home has afforded me the quality sustained time. . .I’ve been able to concentrate [on] things that I’ve had in my head for maybe three years. (FG3)

In addition, some staff described the benefits of home working for allowing them to efficiently fit personal, family responsibilities around work activities. For them, home working helped to balance work and home life. As one participant who was caring for an individual living outside their household stated:

So I’d be able to finish work at five, and get over to his for quarter past five, or at the start it was I had to go and see him at lunchtime. And had I been in the office that would have taken a couple of hours out, probably, rather than 40 minutes. . .So I think there’s been a lot of stuff that people have had to contend with, that working from home has probably made it, in some cases, easier. (INT 8)

### Theme 5: Loss of the social : – Reductions in social connectedness and feelings of isolation

A common reflection was the reduction in social connectedness that occurred because of home working and the restrictions on social contact imposed by the COVID-19 restrictions. Participants described extended periods of home working as a barrier to team cohesion and feelings of connectedness with colleagues. Staff stated that they had missed having face-to-face contact, interaction, and the ability to socialise with work colleagues.

You’re not - you don’t have colleagues around you, you don’t have that rapport with people in the off-. . .like you would in an office. It can be quite lonely. You’re sat in one room all day, often you’re not - you’re speaking to people on the phone, but you’re not having that face-to-face contact with people. So I think I found quite quickly that I much preferred being in the office. . . (INT 9)

During the first few months of the pandemic, videoconferencing technology was implemented as a means for staff to meet and communicate. However, for participants, meeting via videoconferencing technology often felt impersonal and overly ‘business focussed’, with little space for levity and the type of informal, interpersonal interaction that was common in face-to-face meetings prior to the pandemic.

And your Zoom is very focused and ‘business’. And I don’t get to just do that ordinary: ‘what did you do at the weekend? How is your family doing and how are you doing’ That’s something you don’t get to do that, do you?. . .they’ve [staff] lost out perhaps on that social connection, that team connection. And we have a - we try, or you know how you try on Zoom, but it’s not, it’s just not the same, is it. (INT 4)

For some participants, this lack of in-person contact with colleagues resulted in feelings of social isolation which were clearly exacerbated by general restrictions on social contact.

Staff are expressing symptoms of anxiety, tiredness, staff on site are missing the support from having the team around them, feelings of disconnection are expressed. . .some staff at home also missing their colleagues and express feelings of loneliness particularly if living alone. (Timeline entry referring to September 2020).

The pandemic context, COVID-19 restrictions, and the extended period of home working, contributed to some staff feeling isolated at work, and in general, and that this had a detrimental impact on emotional wellbeing of some.

I think every team has had people that have experienced increased anxiety, feelings of isolation, disconnection. . .the feeling of disconnection has definitely created anxiety within the staff teams, despite our innovative working. (FG4)

### Theme 6: The importance of the ‘office’ for team communication and information sharing

Participants highlighted the importance of the office environment and being co-located with others, for building strong working relationships, and facilitating the communication and information sharing viewed as critical to effective delivery of support services. The complex and sensitive nature of drug and alcohol support and the team working that it involves, meant that there was a need for regular contact with colleagues, for example for debriefing, advice and guidance, discussing cases, and gaining emotional support after difficult interactions. Participants stated that a co-located environment was important for enabling the degree of contact, communication and support needed in their roles.

But I think in terms of things that were difficult, you missed your team. . .You’d come off a difficult call in the office, and you’ve got 20 colleagues around you, if you want to speak or vent, or anything like that, and you didn’t have that you were isolated at home. And, fair enough, you could pick up the phone and speak to somebody, but it wasn’t the same. (INT 18)

Another said:

‘You learn from people in the office. You’ve always got your colleagues to sound off to, you’ve always got your colleagues to ask for advice, and there’s all. . . There’s somebody on-hand all the time, where you’re a bit isolated when you’re at home, aren’t you? (INT 2)

It was clear that informal, ad hoc conversations between team members, that took place when staff were co-located, were important for facilitating information-sharing, discussion of client cases, and ultimately for assisting staff in supporting service users. Participants stated that home working had led to a decline in this important type of staff interaction. Reflecting on the contrast between home and office-based working, one participant stated:

So you kind of, you know, and you step away from that [conversation with a service user] and you want to talk with someone who knows what it’s like, your colleague has experienced that. . .Bumping into a prescriber in the kitchen and just going ‘I’ve got this client and I think this, what do you think?’ and have that conversation, rather than having it in your own head and then doubting what’s there. It’s nice to run it past someone. (FG5)

### Theme 7: Managing remotely – The development and implementation of strategies and ways of coping

The shift to home working was challenging for staff and organisations, and our analysis highlighted the development of strategies and practices to support staff in navigating this change and the broader challenges of the COVID-19 pandemic experience. These were apparent at individual, team, and organisational levels.

At an individual level, participants’ coping strategies included, earmarking specific times of day to take breaks and/or devote time to non-work activities, putting firm boundaries around working hours to maintain a clear demarcation between ‘work’ and ‘home’ time, and planning ad hoc visits to the office when feeling particularly isolated working at home.

I think you just have to kind of make a conscious effort to switch off and not be tempted, even if your laptop’s, or your phone is there, to keep it on or anything like that. It was like a conscious effort to keep in a routine as much as you could. (INT 18)

At the team level, staff developed mechanisms for supporting each other when working from home. These included staff setting up informal groups online and via messaging apps to maintain contact and provide peer support; allocating specific times to meet online for informal, social meetings; and managers increasing the frequency with which they ‘checked in’ with staff. As one participant commented:

My team were always really supportive of each other, and we were. . . we’d set up things like WhatsApp groups, and we could have check-in sessions with each other if we needed to. (INT 9)

The lead organisation also acted to support the wellbeing of its employees during the pandemic. This included arranging wellbeing events for staff and encouraging staff to work flexibly when working from home. As one participant commented:

So within that service they’ve set up things like Wellbeing Wednesday, which there was a quiz every Wednesday that you could just join other team members on, and they did stuff like online yoga. (INT 8)

Another said:

Yeah, so, as an organisation, we looked at how we could support people. . .Flexible in how you work, and if you want to log on early, and work a shorter day. If you want to log on late, do that. Don’t stick to your half an hour dinner, please take regular breaks. (INT 17)

## Discussion

This qualitative study explored the home working experiences of drug and alcohol workers directed to work from home during the COVID-19 pandemic. As highlighted in the introduction, although research studies – including some focussing on health and care workers – have examined home-working experiences during the COVID-19 pandemic,^14^ the experiences of those delivering drug and alcohol support services remain under-researched. Given the central role these workers play in addressing the growing burden of substance-related harm and mortality, the distinct nature of their work, and the integration of home working into substance use service delivery since the pandemic, there is a clear need to examine home working in the context of this specific workforce. A clearer understanding of the implications of home working for substance use support staff, their organisations, and those receiving services would help inform decisions about appropriate job design and service delivery models. This study used the example of a team of workers within a single, large local authority area as a case study to explore this research gap.

Home working constituted a significant shift in workers’ typical working practices and impacted their working and home lives. The scope of the change experienced is reflected in the seven themes highlighted, which demonstrate that home working provided various challenges and opportunities which participants navigated using different personal, social, and organisational strategies and resources. As is common in studies of home working,^40,41^ our findings highlighted differing experiences, with both positive and negative experiences represented.

Consistent with previous studies into the experiences of those working from home, our study identified challenges in maintaining a separation and balance between work and home life. Previous research highlights the capacity for home working to result in various challenges of the type described by participants in this study, including blurring of boundaries between work- and home-life; work extension beyond typical working hours; and difficulty disengaging from work.^16,42–47^

Our findings also make a valuable contribution to the home working literature by identifying challenges of home working that may be particular to those employed in roles where upsetting or distressing interactions are common, such as substance use support work. Participants involved in directly supporting service users indicated that home working meant that interactions that would once have been restricted to the office, now took place in their homes – previously personal spaces that had once been a refuge from working life. Mirroring the findings of other studies,^16,48^ we found that such interactions added to the blurring of work and personal spheres, with participants suggesting that the intrusion of these emotionally challenging experiences into the personal space changed the nature of the home environment, and how they felt there. Our findings suggest that, for drug and alcohol support work, it is important to consider the nature of people’s job roles when assessing the appropriateness of home working and of integrating working from home in a hybrid model of service delivery.

It is important to note that research findings about the work-life balance of home workers are equivocal and studies have found that working from home can also have benefits for workers. These include less family-work conflict,^49^ enhanced work-life balance,^45^ increased work flexibility,^50^ less work disturbance,^40^ and greater control over work.^51^ Benefits of home working were also apparent in our study. The ‘convenience and efficiency benefits’ theme presents home working benefits such as reduced travel times and more efficient time management, similar to those identified by previous research.^52–54^ The increased efficiency that can arise from home working has the potential to enhance the quality and responsiveness of drug and alcohol support services.

In keeping with previous research,^46,55,56^ participants expressed feelings of social isolation and a lack of connectedness with colleagues when home working. The unprecedented COVID-19 context and related restrictions undoubtedly contributed to these feelings. However, the communal office environment was explicitly highlighted as important for promoting feelings of team and social connectedness. It was apparent that the requirement to work from home rather than in a communal environment was an important contributor to workers’ feelings of social disconnection and isolation, as noted by previous studies such as Charalampous et al.’s^57^ systematic review of research into the wellbeing of remote workers. This suggests that opportunities for co-located working may be important for supporting the emotional wellbeing of drug and alcohol support workers operating exclusively or predominantly from home.

Participants also indicated that the office environment was important to their ability to deliver a high-quality service. The complex, sensitive nature of drug and alcohol support work necessitated routine emotional support and advice from colleagues, and ad hoc conversations about service users’ cases, which participants said were more effective face-to-face. Maurer et al.’s^58^ case study of communication among teams forced to work from home because of the pandemic lockdown, suggests that imposed home working may have a particularly detrimental impact on communication and cohesion for teams reliant on in-person, ad hoc communication and information sharing. Exclusive home working by drug and alcohol support staff may therefore present a barrier to optimum service delivery because of the challenges it presents to cross-team communication, information sharing, and the opportunity to debrief and receive support.

Previous research has described the processes of change and adaptation experienced by those who transitioned to home working during the pandemic.^21,50,59^ It was apparent from our findings too, that the shift to home working during the pandemic was experienced as a process of adaptation. Participants made incremental changes to their work and home lives to negotiate the new reality of home working in a pandemic context, highlighting the capacity for staff to adapt to new working realities.^60^

Dicu et al.’s^59^ longitudinal qualitative study identified various coping styles adopted by those mandated to work from home. Their study points to changes in coping style over the course of the pandemic, and our study lends support to this idea. Although our data collection was cross-sectional, participants tended to discuss a movement from a situation of uncertainty and disorientation at the start of the pandemic home working period, to one where, after significant negotiation and adaptation, home working was integrated into the work-life cycle, becoming routine, and workable, if not the ideal scenario for all participants.

Personal coping strategies and support from colleagues and organisations can help mitigate the challenges of home working^55,61–63^ and this was reflected in our finding that staff utilised various personal, team, and organisational resources to support their shift to working from home. This included implementing strategies to create separation between work and home life; setting up informal support groups with colleagues; gaining group support through use of technology; and engagement with organisation-wide wellbeing provision. Tong et al.^28^ and Kingstone et al.^48^ are among those who have identified the use of similar strategies during the process of adaptation to home working.

The ways in which staff navigated the demands of home working highlight both the adaptive capacity of drug and alcohol workers and teams, and the key role there may be for employers in supporting transitions to working from home and providing support to sustain effective home working. As Davison^64^ points out, an important notion in the realm of remote working is ‘transformation, not replication’. Our findings suggest that to effectively integrate home and remote working into service delivery, providers of drug and alcohol support services may need to go beyond attempting to replicate pre-pandemic, co-located, office-based ways of working in a home environment. Replication risks transplanting inappropriate or ill-suited practices, rather than identifying the requirements of drug and alcohol staff who work from home and integrating these into home working guidance, policy, and practice.

## Strengths and limitations

The majority of home working research is based on the experiences of those working from home part-time, in relatively stable social environments where home working can be planned.^42,59^ A strength and unique contribution of this study is its focus on neophyte home workers whose experiences were exclusively or predominantly during a global crisis, for whom home working might, pre-pandemic, have been considered especially ill-suited. The study is, to our knowledge, the first specifically exploring the COVID-19 home working experiences of drug and alcohol support workers. It therefore provides new insights that are valuable for informing future service design.

Our study has some limitations. There is the possibility of selection bias in our sample, as staff were asked to voluntarily register to participate rather than being chosen randomly. This may have resulted in systematic differences between our sample and the broader drug and alcohol support worker population. Our sample is also made up of participants from one geographical location, the majority of whom were employed by the same organisation. There is the chance that participants’ experiences may have been significantly coloured by this employing organisation’s employment practices in relation to home working.

In addition, our analysis did not include analysis of the influence of gender on the home working experience. Gender has been shown to be an important factor influencing the experience of home working and home-work conflict.^42,65^

## Conclusion

This study suggests that the experience of delivering drug and alcohol support from home is qualitatively different to delivering it from an employer’s premises, and there may be aspects of drug and alcohol support that are ill-suited to delivery by staff while working from home. To appropriately integrate home working into future substance use service delivery models, it is important that employers are cognisant of the home working experiences of employees and how the needs of home working staff may differ from those of staff working exclusively from their employers’ premises. This study makes an important contribution to this understanding.

Drug and alcohol staff new to homeworking are likely to require support with the transition to working from home. They may also require additional ongoing employer resource, or guidance and protocols for home working similar to those that exist for those working from employer’s premises, particularly where home working is predominant or mandated. Our study suggests that home working may have some benefits for drug and alcohol staff, services and service users. However, the importance of team and multiagency working, effective communication, and staff access to appropriate support to effective service delivery, suggests that exclusive home working by staff is unlikely to be optimal. Our findings suggest that a mix of home and co-located, office-based working, within a ‘hybrid’ (in-person/remote) delivery model is likely to better meet the needs of staff, service users, and service providers.

## Supplemental Material

sj-docx-1-phj-10.1177_22799036251381226 – Supplemental material for Home working during the COVID-19 pandemic: The experience of drug and alcohol support workers Supplemental material, sj-docx-1-phj-10.1177_22799036251381226 for Home working during the COVID-19 pandemic: The experience of drug and alcohol support workers by Nigel Lloyd, Wendy Wills, Imogen Freethy, Olujoke Fakoya, Charis Bontoft, Jaime Garcia-Iglesias, Suzanne Bartington, Gavin Breslin, Neil Howlett, Julia Jones, Katie Newby, Nigel Smeeton, Amander Wellings, David Wellsted and Katherine Brown in Journal of Public Health Research

sj-docx-2-phj-10.1177_22799036251381226 – Supplemental material for Home working during the COVID-19 pandemic: The experience of drug and alcohol support workers Supplemental material, sj-docx-2-phj-10.1177_22799036251381226 for Home working during the COVID-19 pandemic: The experience of drug and alcohol support workers by Nigel Lloyd, Wendy Wills, Imogen Freethy, Olujoke Fakoya, Charis Bontoft, Jaime Garcia-Iglesias, Suzanne Bartington, Gavin Breslin, Neil Howlett, Julia Jones, Katie Newby, Nigel Smeeton, Amander Wellings, David Wellsted and Katherine Brown in Journal of Public Health Research
