# Supplementary material for: Home working during the COVID-19 pandemic: The experience of drug and alcohol support workers
Source: J Public Health Res. 2025 Nov 12;14(4):22799036251381226. doi: 10.1177/22799036251381226 (PMC12612507; doi:10.1177/22799036251381226)
Supplement: sj-docx-1-phj-10.1177_22799036251381226 – Supplemental material for Home working during the COVID-19 pandemic: The experience of drug and alcohol support workers [file sj-docx-1-phj-10.1177_22799036251381226.docx]

Supplementary File 1: COREQ checklist

Consolidated criteria for reporting qualitative studies (COREQ): 32-item checklist

Developed from:

Tong A, Sainsbury P, Craig J. Consolidated criteria for reporting qualitative research (COREQ): a 32-item checklist for interviews and focus groups. International Journal for Quality in Health Care. 2007. Volume 19, Number 6: pp. 349 – 357

| **Item No** | | **Guide Questions/Description** | **Reported on Page #** |  |
| --- | --- | --- | --- | --- |
| **Domain 1: Research team and reflexivity** | | | |  |
| **Personal Characteristics** | | | |  |
| 1. Interviewer/ facilitator | | Which author/s conducted the interview or focus group? | This is stated in the ‘contribution’ section.  NL, CB, IF, JGI and KB conducted interviews and focus groups.  NL and JGI led the digital timeline tool development and data collection, with significant contribution from WW. |  |
| 2. Credentials | | What were the researcher’s credentials? E.g., PhD, MD | Nigel Lloyd (MA) – researcher with more than 29 years’ continuous research and evaluation  expertise;  Wendy Wills (Professor, PhD);  Imogen Freethy (BSc);  Olujoke Fakoya (PhD);  Charis Bontoft (MSc);  Jaime Garcia-Iglesias (PhD);  Suzanne Bartington (PhD);  Gavin Breslin (PhD);  Neil Howlett (PhD);  Julia Jones(PhD);  Katie Newby (PhD);  Nigel Smeeton (MSc);  Amander Wellings (MA);  David Wellsted (PhD);  Katherine Brown (Professor, PhD). |  |
| 3. Occupation | | What was their occupation at the time of the study? | Nigel Lloyd (Senior Research Fellow);  Wendy Wills (Professor);  Imogen Freethy (Research Assistant);  Olujoke Fakoya (Research Fellow);  Charis Bontoft (Research Assistant);  Jaime Garcia-Iglesias (Research Fellow);  Suzanne Bartington (Associate Professor);  Gavin Breslin (Senior Lecturer);  Neil Howlett (Senior Research Fellow);  Julia Jones (Professor);  Katie Newby (Associate Professor);  Nigel Smeeton (Social Statistician);  Amander Wellings (PPI co-applicant);  David Wellsted (Reader);  Katherine Brown (Professor). |  |
| 4. Gender | | Was the researcher male or female? | The research team was a mix of male and female researchers. |  |
| 5. Experience and training | | What experience or training did the researcher have? | The research team had extensive expertise and training in research and evaluation across many dozens of projects.  For example:   - NL has extensive research training, more than 29 years’ experience of mixed-methods research and evaluation, and has successfully managed more than 50 research and evaluation projects. - KB has more than 20 years’ experience of mixed methods applied health research and is a highly-regarded Professor of Public Health; - JGI has a PhD in Sociology and expertise in qualitative research methods. - OF has a PhD and expertise in qualitative research methods. - IF and CB were graduate research assistants at the time of the study and were trained and supervised by the wider research team throughout. - Wendy Wills is Professor of Food and Public Health, with decades of research experience.   Details on the credentials of other team members is available on request. |  |
| **Relationship with participants** | | | |  |
| 6. Relationship established | | Was a relationship established prior to study commencement? | No prior relationship was established between the researchers and participants prior to study commencement, apart from some preliminary awareness raising across the service delivering organization about the forthcoming evaluation. |  |
| 7. Participant knowledge of the interviewer | | What did the participants know about the researcher? e.g. personal goals, reasons for doing the research? | Participants were aware of the nature and purpose of the study and that it was an academic research study funded by the National Institute of Health Research and conducted in partnership with local government and their organisations. Participants were not made aware of any personal researcher goals or motivations. |  |
| 8. Interviewer characteristics | | What characteristics were reported about the interviewer/facilitator? e.g. Bias, assumptions, reasons and interests in the research topic | As above, participants were provided with a ‘participant information sheet’ outlining the nature and purpose of the study, funding, what involvement would entail, and ethical considerations such as how data would be recorded and stored and for how long, the voluntary nature of involvement, and the right to withdraw participation. Participants were not made aware of any researcher personal motivations or interest in the topic. |  |
| **Domain 2: study design** | | |  |  |
| **Theoretical framework** | | |  |  |
| 9. Methodological orientation and Theory | What methodological orientation was stated to underpin the study? e.g. grounded theory, discourse analysis, ethnography, phenomenology, content analysis | The study took a broadly phenomenological approach to capture the common features of the phenomenon under study. Our qualitative analysis is underpinned by a broad constructivist/interpretivist orientation which acknowledges the role of individuals’ experiences and interpretations in framing their subjective, constructed realities. This has been clarified on page 9. |  |  |
| **Participant selection** | | |  |  |
| 10. Sampling | How were participants selected? e.g., purposive, convenience, consecutive, snowball | This is reported in the ‘Recruitment and sampling’ section (pages 9-10). Additional text has been added to clarify. |  |  |
| 11. Method of approach | How were participants approached? e.g., face-to-face, telephone, mail, email | This is reported in the ‘Recruitment and sampling’ section (pages 9-10). |  |  |
| 12. Sample size | How many participants were in the study? | As stated in the ‘participants’ section (page 11), the analysis presented is based on data from 30 participants. |  |  |
| 13. Non-participation Setting | How many people refused to participate or dropped out? Reasons? | On page 10, in the ‘Recruitment and sampling’ section, further detail has been added about the number of those invited to participate who subsequently did so. Information is provided for each data collection method, which also provides additional information about sampling methods. |  |  |
| 14. Setting of data collection | Where was the data collected? e.g., home, clinic, workplace | Participants took part remotely during their regular working hours, either from their workplace or while working from home. Clarification has been added on page 9. |  |  |
| 15. Presence of nonparticipants | Was anyone else present besides the participants and researchers? | No, only the researchers and participants were present during data collection. |  |  |
| 16. Description of sample | What are the important characteristics of the sample? e.g. demographic data, date | To limit the collection of personal data to only what was strictly necessary for the study, information on age and gender was not collected. However, all participants were aged 18 years or older and various study-relevant participant characteristics were requested, such as, whether they had worked remotely since the start of the COVID-19 pandemic; time in job role; and nature of role (e.g., management, administrative or frontline service delivery). These details are provided in the ‘Participants’ section (page 11). |  |  |
| **Data collection** | | |  | No |
| 17. Interview guide | Were questions, prompts, and guides provided by the authors? Was it pilot tested? | Yes, interview and focus group schedules were used, and an online timeline template was provided for timeline respondents, as stated in the ‘Procedure’ section. A copy of the interview/focus group schedule has been provided as a supplementary document.  The schedules and online timeline were piloted with our Public Involvement in Research Group (PIRg) prior to commencement of data collection to ensure that the research tools were appropriate and would enable us to collect data that would address our research questions. |  |  |
| 18. Repeat interviews | Were repeat interviews carried out? If yes, how many? | None were conducted. |  |  |
| 19. Audio/visual recording | Did the research use audio or visual recording to collect the data? | Yes, this is stated in the ‘Procedure’ section (page 12). |  |  |
| 20. Field notes | Were field notes made during and/or after the interview or focus group? | No, no field notes were not routinely made. |  |  |
| 21. Duration | What was the duration of the interviews or focus group? | Details on interview and focus group duration and timeline availability are provided in the ‘‘Procedure’ section (pages 11-12). |  |  |
| 22. Data saturation | Was data saturation discussed? | Yes, ‘data saturation’ is discussed - ‘thematic saturation’ was reached prior to ceasing our analysis as outlined on page 14. |  |  |
| 23. Transcripts returned | Were transcripts returned to participants for comment and/or correction? | No, transcripts were not returned but participants were informed that they could view their own transcript should they wish to do so. |  |  |
| **Domain 3: analysis and findings** | | |  |  |
| **Data analysis** | | |  |  |
| 24. Number of data coders | How many data coders coded the data? | Six members of the research team were involved in coding as indicated in the ‘Data coding and analysis’ section (pages 12 and 13). |  |  |
| 25. Description of the coding tree | Did the authors provide a description of the coding tree? | No, this has not been provided. |  |  |
| 26. Derivation of themes | Were themes identified in advance or derived from the data? | Both inductive and deductive coding was conducted, with themes then derived from the data during the mapping and interpretation components of our Framework analysis, as outlined on pages 13-14. |  |  |
| 27. Software | What software, if applicable, was used to manage the data? | As stated in the ‘Data coding and analysis’ section (page 12), online timeline text was treated as qualitative text-based data, and as with the interview and focus group transcripts, was coded using NVivo 12 software. |  |  |
| 28. Participant checking | Did participants provide feedback on the findings? | No, participants did not provide feedback. |  |  |
| **Reporting** | | |  |  |
| 29. Quotations presented | Were participant quotations presented to illustrate the themes/findings? Was each quotation identified? e.g., participant number | Yes, participant quotations are presented to illustrate the themes/findings throughout, with participant ID codes used for quotes (pages 14 to 25). |  |  |
| 30. Data and findings consistent | Was there consistency between the data presented and the findings? | Yes, there is consistency between the data and the findings. The paper clearly indicates how the data leads to and relates to the findings. |  |  |
| 31. Clarity of major themes | Were major themes clearly presented in the findings? | Yes, major themes are clearly presented and discussed in detail in the Results section (pages 14 to 25). |  |  |
| 32. Clarity of minor themes | Is there a description of diverse cases or a discussion of minor themes? | Sub-themes are not explicitly identified; instead, each major theme is explored in depth. Rather than offering a cursory overview, we have aimed to provide a nuanced discussion that examines the underlying concepts and meanings of each theme (pages 14 to 25). |  |  |
