# Supplementary material for: Home working during the COVID-19 pandemic: The experience of drug and alcohol support workers
Source: J Public Health Res. 2025 Nov 12;14(4):22799036251381226. doi: 10.1177/22799036251381226 (PMC12612507; doi:10.1177/22799036251381226)
Supplement: sj-docx-2-phj-10.1177_22799036251381226 – Supplemental material for Home working during the COVID-19 pandemic: The experience of drug and alcohol support workers [file sj-docx-2-phj-10.1177_22799036251381226.docx]

**Schedule for one-to-one interviews/focus groups with staff members**

**Could you explain what your job is and how long you have been doing it?**

Probe:

- What ‘team’ do you work within? How large is the ‘team’?
- General nature of day-to-day role?
- What specific service user groups (including, substance use categories) do you typically work with?

**In what way/s, if at all, did your job change during the COVID-19 pandemic?**

Probe:

- - What did your role involve before COVID-19? Caseload?
  - What have been the main changes to how you work?
  - Changes in nature of work, focus of work, caseload?
  - Were there aspects that changed from face-to-face to remote and what is/was the nature of this change?

**When and how were you told about these changes? How did you feel about them initially?**

**Where do you work from these days (how has this changed as a result of Covid-19)?**

Probe:

- Change during the pandemic?
- What has the mix of home- working/working in or at employers’ premises (or working in a more traditional sense) been like?

**In general terms, how has COVID-19 impacted your working life?**

Probe:

- General description? General overview of positive and negative aspects?
- Wider life outside of work?

**What support have you received from your employer around changes to your working life/the way that you work?**

Probe:

- Adequacy of support? What more might have been beneficial?

**Could you tell me more about your experience of working from home?**

Probe:

- How long did it take you to get set up?
- How has IT/connectivity/wi-fi been?
- How did you choose where to work from within your home?
- How has it been/was it managing important or personal meetings from home?
- What, if anything, have you done/did you do to make this work better?

**How have your home life and responsibilities impacted on any homeworking you’ve had to do?**

Probe:

- Impact of caring responsibilities (e.g. childcare or family members)?

**Overall, how/has is working from home going/gone for you?**

Probe:

- How do you find separating work and home life while working at home?
- Any particular challenges? How have you managed these?
- Anything you particularly like or feel is beneficial?
- What, if anything do you miss about the way you worked before COVID-19 work?

**How do you feel about going back to working as you were prior to COVID-19 (e.g., back to the office)?**

Probe:

- Anything you are particularly looking forward to or not looking forward

to?

**How, if at all, did the changes to the way you worked during the pandemic affect your relationships with your colleagues?**

Probe:

- Relationships with managers, colleagues, peers?
- How were these affected and why?
- Did this affect service delivery?

**Conclusion and debrief**

- Is there anything else you’d like to add?
- Were you expecting me to ask anything else that we haven’t covered?
- Participant is thanked for their participation and asked if they have any questions.
- How the data will be used will is restated.
